# Highly Thermally Conductive and Flame-Retardant Waterborne Polyurethane Composites with 3D BNNS Bridging Structures via Magnetic Field Assistance

**DOI:** 10.1007/s40820-025-01651-1

**Published:** 2025-02-07

**Authors:** Hao Jiang, Yuhui Xie, Mukun He, Jindao Li, Feng Wu, Hua Guo, Yongqiang Guo, Delong Xie, Yi Mei, Junwei Gu

**Affiliations:** 1 Yunnan Provincial Key Laboratory of Energy Saving in Phosphorus Chemical Engineering and New Phosphorus Materials, The International Joint Laboratory for Sustainable Polymers of Yunnan Province, The Higher Educational Key Laboratory for Phosphorus Chemical Engineering of Yunnan Province, Faculty of Chemical Engineering, Kunming University of Science and Technology, Kunming, 650500 People’s Republic of China; 2 Shaanxi Key Laboratory of Macromolecular Science and Technology, School of Chemistry and Chemical Engineering, Northwestern Polytechnical University, Xi’an, 710072 People’s Republic of China

**Keywords:** Boron nitride nanosheets, Magnetic response, Structural design, Thermal conductivity, Flame retardancy

## Abstract

The microstructure design for thermal conduction pathways in polymeric electrical encapsulation materials is essential to meet the stringent requirements for efficient thermal management and thermal runaway safety in modern electronic devices. Hence, a composite with three-dimensional network (Ho/U-BNNS/WPU) is developed by simultaneously incorporating magnetically modified boron nitride nanosheets (M@BNNS) and non-magnetic organo-grafted BNNS (U-BNNS) into waterborne polyurethane (WPU) to synchronous molding under a horizontal magnetic field. The results indicate that the continuous in-plane pathways formed by M@BNNS aligned along the magnetic field direction, combined with the bridging structure established by U-BNNS, enable Ho/U-BNNS/WPU to exhibit exceptional in-plane (*λ*_*//*_) and through-plane thermal conductivities (*λ*_**⊥**_). In particular, with the addition of 30 wt% M@BNNS and 5 wt% U-BNNS, the *λ*_*//*_ and *λ*_**⊥**_ of composites reach 11.47 and 2.88 W m^−1^ K^−1^, respectively, which representing a 194.2% improvement in *λ*_**⊥**_ compared to the composites with a single orientation of M@BNNS. Meanwhile, Ho/U-BNNS/WPU exhibits distinguished thermal management capabilities as thermal interface materials for LED and chips. The composites also demonstrate excellent flame retardancy, with a peak heat release and total heat release reduced by 58.9% and 36.9%, respectively, compared to WPU. Thus, this work offers new insights into the thermally conductive structural design and efficient flame-retardant systems of polymer composites, presenting broad application potential in electronic packaging fields.

**Supplementary Information:**

The online version contains supplementary material available at 10.1007/s40820-025-01651-1.

## Highlights

- By simultaneously incorporating the magnetic filler-modified boron nitride nanosheets (M@BNNS) and the non-magnetic filler U-BNNS into the polymer matrix, a three-dimensional heat conduction pathway composites are obtained under a horizontal magnetic field.
- Owing to the microstructural design of the 3D-bridging architecture, with the addition of only 5 wt% U-BNNS, the *λ*_**⊥**_ of composites achieved 2.88 W m^−1^ K^−1^, representing a remarkable increase of 194.2% compared to single-oriented composites.
- The 3D-bridging architecture composite also demonstrates excellent flame retardancy, attributed to the synergistic mechanisms of condensed and gas phases, effectively mitigating the risks of thermal runaway in electronic devices.

**Supplementary Information:**

The online version contains supplementary material available at 10.1007/s40820-025-01651-1.

## Graphical abstract

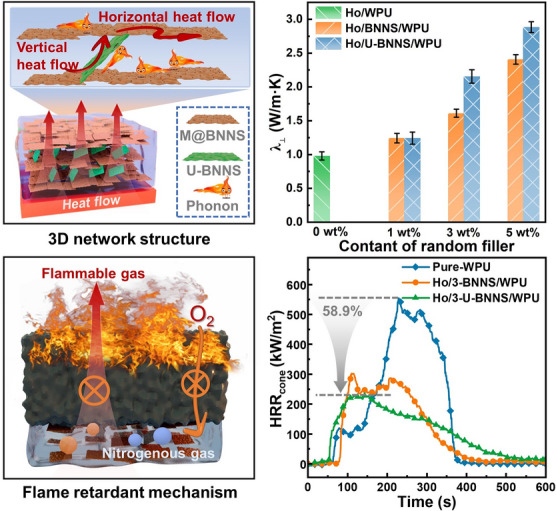

## Introduction

The rapid development of miniaturized and integrated electronic devices has led to increasing power densities and heat generation, causing internal heat accumulation that poses significant threats to the reliability and safety of electronic systems [1–3]. This challenge has heightened the demand for efficient thermal management and the mitigation of thermal runaway risks in electronic packaging materials [4, 5]. Polymers are widely employed in the encapsulation of electronic components due to their excellent insulation, ease of processing, and chemical stability [6, 7]. However, traditional polymers suffer from inherently low thermal conductivity, limited thermal stability, and flammability, restricting their application in advanced electronic devices [8–10]. To overcome these limitations, a widely adopted strategy is the incorporation of functional fillers, such as aluminum oxide [11], aluminum nitride [12], and boron nitride [13], into the polymer matrix, significantly advancing the development of polymer-based thermal management materials. Among these, two-dimensional (2D) materials like MXene and graphene have been extensively studied for their unique ability to enhance both the thermal conductivity and flame retardancy of polymer composites [14, 15]. However, the inclusion of large amounts of conductive fillers such as MXene and graphene would compromise the insulation properties of the polymer matrix, increasing the likelihood of electrical short circuits in precision electronic packaging and consequently reducing the overall reliability of electronic devices [16, 17].

Boron nitride nanosheets (BNNS), derived from the exfoliation of hexagonal boron nitride (*h*-BN), are a graphene-like 2D material celebrated for their exceptional in-plane thermal conductivity, electrical insulation, and thermal stability [18, 19]. These properties make BNNS highly promising for applications in thermal management materials for electronic devices. Yang et al. [20] developed surface-modified BNNS through ball milling and hyperbranched surface modification, subsequently incorporating them into epoxy resin to fabricate thermally conductive composites. With a loading of 20 wt% modified BNNS, the composite achieved a through-plane thermal conductivity (*λ*_**⊥**_) of 0.88 W m^−1^ K^−1^, maintaining excellent electrical insulation properties. Wang et al. [21] employed liquid-phase film processing to fabricate BNNS, mixing it with polydimethylsiloxane to create a thermally conductive composite. At a BNNS loading of 4 wt%, the composite exhibited an in-plane thermal conductivity (*λ*_*//*_) of 4.69 W m^−1^ K^−1^ and *λ*_**⊥**_ of 0.23 W m^−1^ K^−1^. The structural characteristics of BNNS enable theoretically *λ*_*//*_ to reach up to 1000–2000 W m^−1^ K^−1^, whereas its *λ*_**⊥**_ is significantly lower (approximately 30 W m^−1^ K^−1^), which highlights its anisotropic thermal properties [22, 23]. However, when BNNS is randomly dispersed within a polymer matrix, its effectiveness in enhancing thermal conductivity is limited [1, 24]. Therefore, controlling the alignment of BNNS within the matrix to establish continuous thermally conductive pathways is crucial for maximizing its thermal conductivity enhancement potential, ultimately leading to substantial improvements in the thermal performance of polymer composites.

Currently, common methods for constructing BNNS-based thermally conductive microstructures include hot-pressing, ice-templating, sacrificial templating, and magnetic field orientation techniques [25, 26]. In the hot-pressing approach, the polymer chain segment is heated and stretched in the direction of pressure. The 2D fillers align along the plane parallel to the pressure, due to the difference in extensibility between the polymer matrix and the rigid filler. And the directional thermal conductivity of composites is enhanced [27]. For instance, Zhou et al. [28] used dopamine-modified BNNS in polyvinyl alcohol to prepare horizontally aligned composites through hot pressing. With a BNNS content of 35.5 wt%, the composite achieved a *λ*_*//*_ of 16.6 W m^−1^ K^−1^. However, this method is mainly effective for constructing horizontally aligned structures and less suitable for achieving high *λ*_**⊥**_ due to its limitations in creating vertical microstructures. Ice-templating and sacrificial templating methods involve constructing a 3D thermally conductive framework from the filler, followed by infusion of the polymer matrix to form the composite [29]. For example, Liang et al. [30] employed ice-templating to create silicone composites with continuous thermal pathways at ultra-low BNNS loading (0.85 vol%), achieving a *λ*_**⊥**_ of 1.54 W m^−1^ K^−1^. However, these templating methods involve multiple steps, such as pre-fabrication of the thermal conductive framework followed by polymer infusion, making the process complex and challenging for industrial-scale applications. By contrast, magnetic alignment techniques offer a more streamlined approach. This method enables the alignment of magnetically responsive fillers within the matrix while in solution form, and the composite can be simultaneously molded. This one-step process is particularly advantageous for enhancing thermal conductivity in a specific direction by aligning 2D fillers under a parallel magnetic field [31]. He et al. [32] developed magnetically responsive fillers by self-assembling *h*-BN and ferrites, which are then mixed with polyvinylpyrrolidone and molded under a parallel magnetic field. The resulting composite, with a filler content of 62.6 vol%, exhibited a *λ*_**⊥**_ of 12 W m^−1^ K^−1^. However, magnetic alignment methods typically enhance thermal conductivity in a single direction, while thermal conductivity perpendicular to the alignment direction often remains lower than that of composites with randomly distributed fillers [33]. This is because the 2D fillers form continuous thermal pathways only along the magnetic field direction, with insufficient connectivity in the perpendicular direction, reducing overall thermal efficiency. Thus, the development of composites with 3D thermally conductive microstructures using magnetic alignment techniques, capable of enhancing thermal conductivity in multiple directions simultaneously, remains a significant challenge.

To address the above challenge, this work employed a composite with three-dimensional network (Ho/U-BNNS/WPU) that is developed by simultaneously incorporating magnetically modified boron nitride nanosheets (M@BNNS) and non**-**magnetic organo-grafted BNNS (U-BNNS) into waterborne polyurethane (WPU) to synchronous molding under a horizontal magnetic field. The result demonstrated that the alignment of M@BNNS along the magnetic field direction formed a continuous horizontal pathway; meanwhile, U-BNNS created a bridging structure between these horizontal pathways. This architecture endowed Ho/U-BNNS/WPU with excellent in-plane and through-plane heat conductivities. Furthermore, the composite exhibited remarkable flame retardancy. In practical applications of LED and chips, the composites also demonstrated outstanding thermal management capabilities. Therefore, this work provides novel insights into the structural design of thermally conductive composites and highlights their potential for thermal management in integrated circuits and batteries.

## Experimental Section

### Materials

Hexagonal boron nitride powder (*h*-BN, particle size: 5–10 μm, 99.9%), hexamethylene diisocyanate (HDI, 97%), 6-methylisocytosine (MIC, 99.5%), and dibutyltin dilaurate (99%) are acquired from Shanghai Aladdin Bio-Chem Technology Co., Ltd. Waterborne polyurethane is sourced from Anhui Huatai New Materials Co., Ltd. Ethanol (EtOH, analytical grade), ethylene glycol (analytical grade), sodium hydroxide (97%), potassium hydroxide (95%), manganese chloride tetrahydrate (99.9%), ferric chloride hexahydrate (99%), ammonia solution (28 wt%), polyvinylpyrrolidone (PVP, molecular weight: 8000, 99%), and urea (99%) are obtained from Shanghai Macklin Biochemical Co., Ltd.

### Preparation of Functional Fillers

The fillers preparation process consists of three main stages: exfoliation of h-BN, magnetic modification of boron nitride nanosheets (M@BNNS), and 2-ureido-4[1H]-pyrimidinone (UPy) modification of boron nitride nanosheets (U-BNNS). First, bulk h-BN underwent high-temperature hydrothermal treatment with a mixed alkaline solution, followed by exfoliation using a high-speed shear machine to produce BNNS. Next, the obtained BNNS is combined with manganese chloride tetrahydrate, manganese chloride hexahydrate, urea, and PVP in an ethylene glycol solution. The mixture is adjusted to a weakly alkaline level using an ammonia solution. After hydrothermal treatment, the manganese ferrite in situ composite M@BNNS is synthesized. The preparation of U-BNNS involved thoroughly mixing BNNS with self-synthesized UPy in DMF. In this process, the isocyanate end group of UPy reacts with the hydroxyl groups on the surface of BNNS, resulting in the grafting of UPy onto the BNNS surface and yielding U-BNNS. Further details for the preparation of functional fillers can be found in the Supporting Information S1 Experimental Section.

### Preparation of Polymer Composite with Different Structures

The 3D-bridge structured composites are prepared by simultaneously incorporating magnetic nanosheets (M@BNNS) and non**-**magnetic nanosheets (U-BNNS) into WPU to synchronous molding under a horizontal magnetic field. For instance, the composite containing 30 wt% M@BNNS and 3 wt% U-BNNS (designated as Ho/3-U-BNNS/WPU) is created. In this process, 3 g of M@BNNS powder, 0.3 g of U-BNNS powder, and 16.75 g of WPU dispersion (40% solid content) are mixed in a round-bottom flask. The mixture undergoes simultaneous mechanical stirring and ultrasonic treatment for 60 min to ensure homogeneity. After 5 min of vacuum degassing, the mixture is poured into a polytetrafluoroethylene mold for shaping. The mold is placed on a custom-made rotating platform set to 5 rpm. Two N52 magnets are positioned on either side of the rotating platform, with the distance adjusted to maintain a magnetic field strength of 30 to 50 mT at the center of the platform. This arrangement allows M@BNNS to align effectively within the matrix. After stopping the rotation, the mixed solution is allowed to dry naturally, resulting in the composites Ho/3-U-BNNS/WPU. By adjusting the filler that is unaffected by the magnetic field while keeping other conditions constant, various composites can be produced, such as the 3D-bridge structured composite containing 3 wt% BNNS, designated as Ho/3-BNNS/WPU. The detailed compositions of samples are provided in Table S1. For detailed procedures regarding the synthesis of UPy, U-BNNS, and the composites, as well as the instruments and characterization methods used, please refer to Supplementary Information S1 Experimental Section.

## Results and Discussion

### Preparation and Characterization of Fillers

**Fig. 1:**
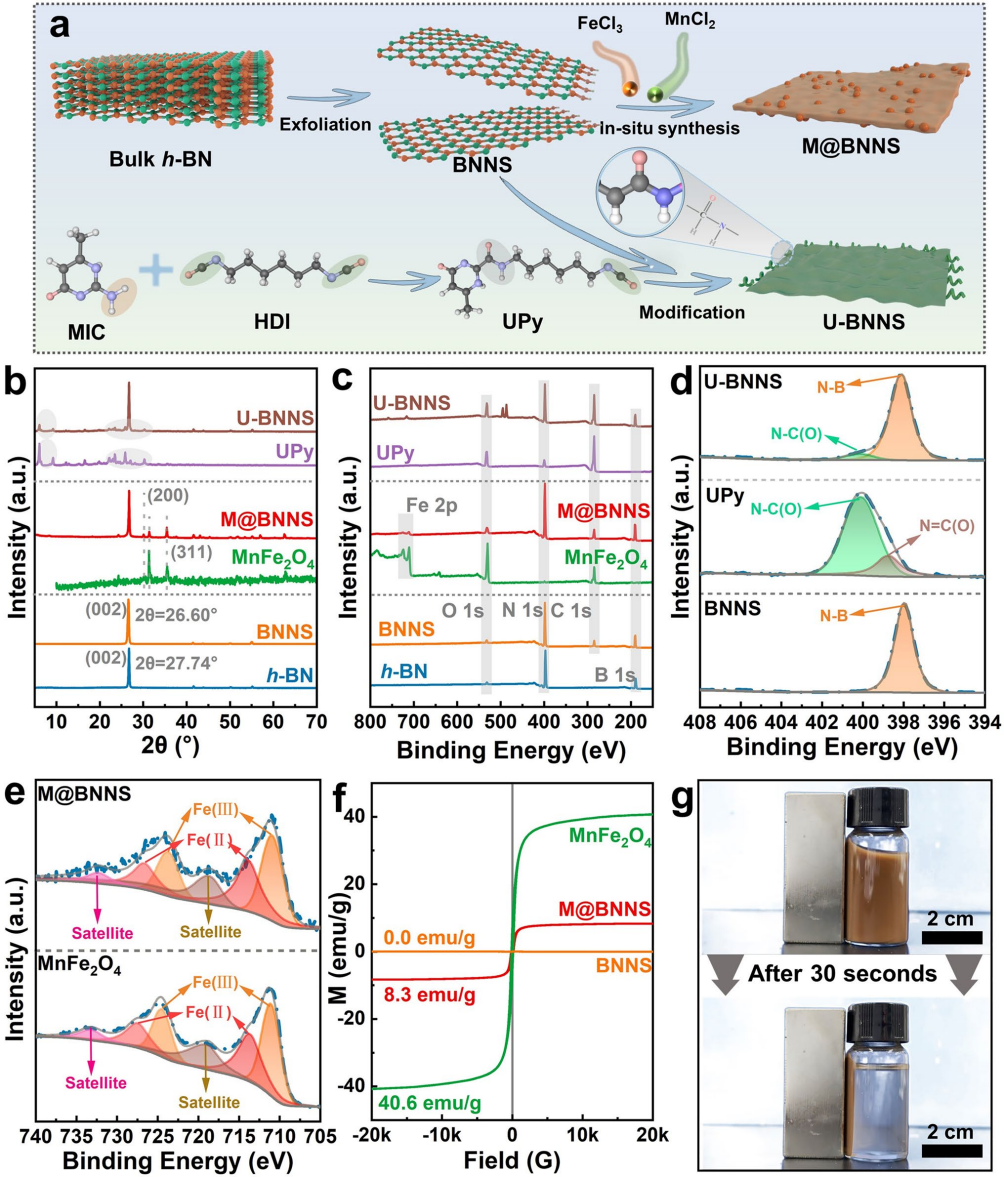
**a** Preparation diagram of M@BNNS and U-BNNS; surface structure and property characterization of materials at each stage of h-BN stripping and modification process, **b** XRD, **c** XPS survey spectrum, **d** XPS high-resolution spectra of N 1*s*, and **e** XPS high-resolution spectra of Fe 2*p*; **f** hysteresis curves of BNNS, M@BNNS, and MnFe_2_O_4_; **g** M@BNNS photographs of magnet adsorption experiments on suspension

The preparation process of multiple functional fillers, including BNNS, M@BNNS, and U-BNNS, is illustrated in Fig. 1. A magnetically responsive 2D material, M@BNNS, is synthesized via in situ hydrothermal deposition of MnFe_2_O_4_ on the surface of BNNS. Concurrently, a non-magnetically responsive 2D material, U-BNNS, featuring hydrogen-bonding interactions, is prepared through a reaction between the isocyanate groups of UPy and the hydroxyl groups on the BNNS surface. Furthermore, Fig. 1b-g presents the surface structure and compositional characterization of the functional fillers involved in the exfoliation and functionalization of BNNS. As shown in Fig. 1b, the X-ray diffraction (XRD) pattern reveals a blue shift of the (002) characteristic peak of BNNS from 27.74° to 26.60°, indicating a significant increase in interlayer spacing compared to h-BN after hydrothermal treatment and shear exfoliation. This reduction in interlayer interaction confirms the successful exfoliation of BNNS [34]. Further analysis of the XRD spectrum for M@BNNS shows not only the (002) peak of BNNS but also characteristic peaks at (311) and (200) corresponding to MnFe_2_O_4_ [35], verifying the successful synthesis of M@BNNS. In U-BNNS, the characteristic peaks corresponding to UPy are observed, as indicated in the gray section of the figure, confirming the composite formation of UPy with BNNS [36]. Additionally, U-BNNS is synthesized through the reaction between the isocyanate groups of UPy and the hydroxyl groups of BNNS. Figure S1 displays Fourier transform infrared (FTIR) and ^1^H NMR spectra of synthesized UPy. The absorption peaks at 2936, 2860, and 2275 cm⁻^1^ correspond to -CH₃, -CH₂, and -NCO groups of HDI, respectively [37]. The peak at 3336 cm⁻^1^ for MIC arises from the N–H stretching vibration of the pyridine ketone ring. The UPy spectrum includes all these characteristic peaks, along with a peak at 1703 cm⁻^1^ corresponding to the carbonyl group (-NH-CO–NH) of the urea, and another at 1256 cm⁻^1^ for the C-N bond in the urea, confirming the successful synthesis of UPy and indicating that the retained -NCO groups facilitate subsequent grafting reactions with the hydroxyl groups of BNNS [38]. Furthermore, the ^1^H NMR spectrum of UPy shown in Fig. S1b illustrates the main functional groups corresponding to the chemical shifts of the peaks [39], further validating the successful synthesis of UPy, which subsequently reacts with the hydroxyl groups of BNNS to form U-BNNS.

X-ray photoelectron spectroscopy (XPS) is employed to investigate changes in surface elemental composition and structure during the modification of BNNS, as shown in Fig. 1c-e and detailed in Table S2. The survey XPS spectrum indicates that following MnFe_2_O_4_ modification, the surface of BNNS exhibits characteristic peaks corresponding to Mn 2*p* and Fe 2*p*. Additionally, the U-BNNS sample reveals a significantly higher content of N and O compared to unmodified BNNS, confirming alterations in the elemental composition due to the modification process. Further analysis of the N 1*s* high-resolution spectra for BNNS, UPy, and U-BNNS is presented in Fig. 1d. Characteristic peaks for UPy at 398.78 and 400.18 eV correspond to the N = C(O) and N–C(O) groups, respectively [36]. Notably, the N elements in modified U-BNNS appear at 398.08 and 400.18 eV, corresponding to N-B and N–C(O) groups, while the peak for N = C(O) disappears [22]. This indicates that the isocyanate groups of UPy reacted with the hydroxyl groups on the surface of BNNS during preparation, successfully yielding U-BNNS. Figure 1e illustrates the Fe 2*p* high-resolution spectra for MnFe_2_O_4_ and M@BNNS. The MnFe_2_O_4_ shows characteristic peaks at 711.18, 713.78, 724.38, and 727.48 eV, corresponding to Fe(II) 2*p*_1/2_, Fe(III) 2*p*_1/2_, Fe(II) 2*p*_3/2_, and Fe(III) 2*p*_3/2_, along with two satellite peaks at 719.08 and 733.08 eV, indicating the presence of iron in two oxidation states [40]. Similarly, the surface of BNNS after hydrothermal treatment with MnFe_2_O_4_ exhibits six characteristic peaks that correspond to those of MnFe_2_O_4_, confirming the successful preparation of M@BNNS.

To further assess the magnetic responsiveness of the modified M@BNNS, the hysteresis loop of the sample is measured, as shown in Fig. 1f. The saturation magnetization of MnFe_2_O_4_ is found to be 40.6 emu g^−1^, which is consistent with the reports in the literature [41]. And, the saturation magnetization of unmodified BNNS exhibits 0 emu g^−1^, indicating a lack of magnetic responsiveness. In contrast, the modified M@BNNS exhibits a saturation magnetization of 8.3 emu g^−1^, indicating a certain level of magnetic response. Figure 1g and Video S1 illustrate a magnetic attraction experiment with M@BNNS suspension. Here, the brown M@BNNS gradually aggregates toward the magnet under the influence of the magnetic field, completely adhering to the magnet after 30 s, while the solution transitions from brown to clear. This experiment provides a clear demonstration of the magnetic responsiveness of M@BNNS.

**Fig. 2:**
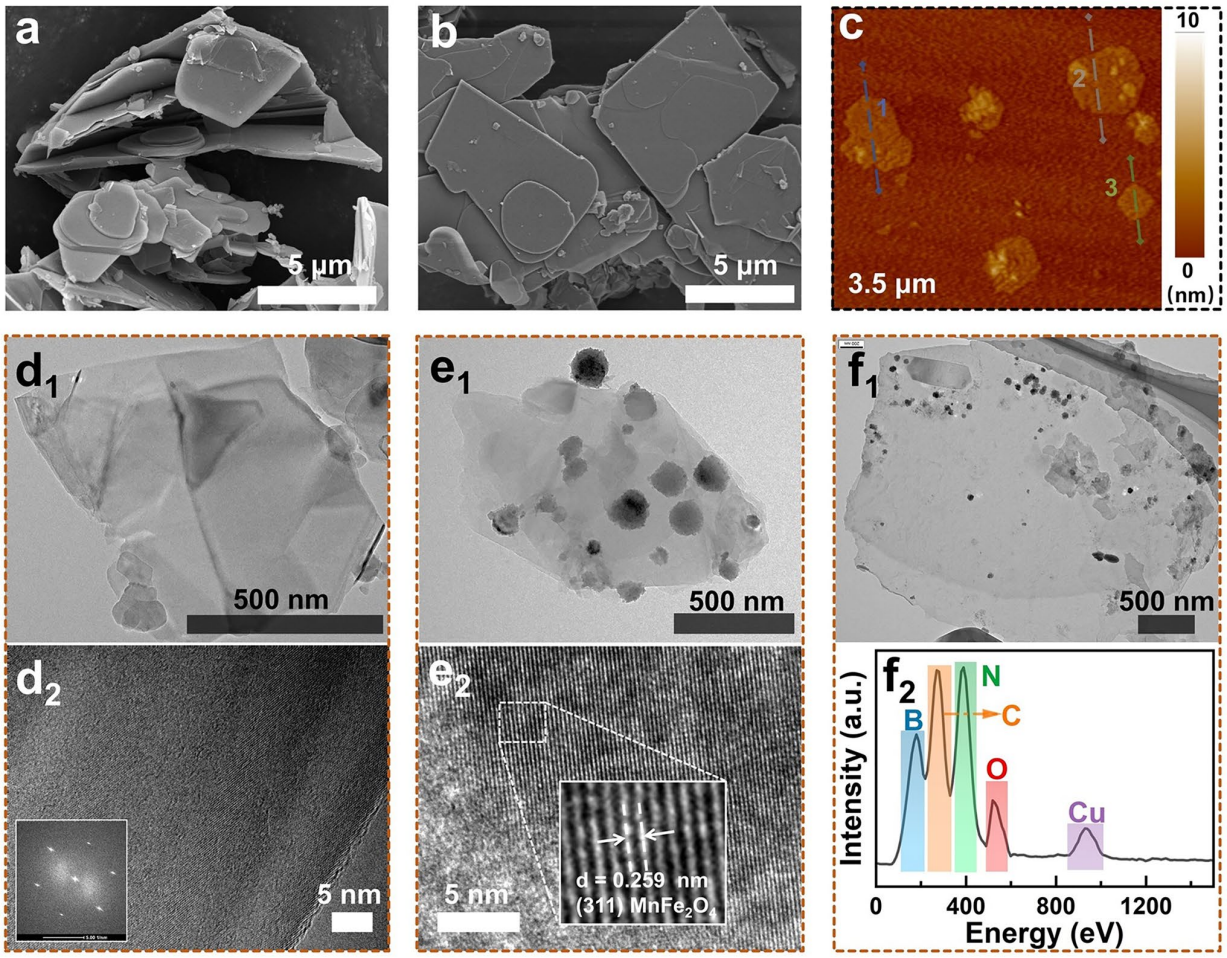
**a** SEM image of bulk *h*-BN; **b** SEM images, **c** AFM images, and **d** TEM images of BNNS; **e** TEM images of M@BNNS and the corresponding high-resolution images; **f** TEM images of U-BNNS, and matching EDS element analysis

Figure 2 illustrates the effects of the peeling and modification processes of *h*-BN on its microstructure and surface characteristics. The scanning electron microscope (SEM) image in Fig. 2a shows that the original h-BN exhibits lateral dimensions ranging from 2 to 10 μm and a thickness of 200 to 500 nm, characterized by tightly stacked layers. In contrast, as depicted in Fig. 2b-d, the surface morphology of BNNS indicates a significant reduction in flake thickness after hydrothermal treatment and shear exfoliation, with surface wrinkling indicating the successful formation of a thin-sheet structure. The atomic force microscopy analysis (Fig. S2) further confirms that the flake thickness of BNNS is approximately 2 to 3 nm, indicating a few-layered nanosheet structure. The transmission electron microscopy (TEM) image of BNNS in Fig. 2d shows high transparency and a few-layer nanosheet structure, with distinct lattice fringes and clear hexagonal diffraction spots, indicating that BNNS retains its crystalline structure, essential for rapid phonon diffusion and excellent thermal conductivity [42]. To assess the impact of MnFe_2_O_4_ modification, the TEM characterization of M@BNNS is shown in Fig. 2e, where spherical MnFe_2_O_4_ particles, ranging from 100 to 300 nm, are observed on the surface of BNNS nanosheets. High-resolution TEM images reveal lattice fringes measuring 0.26 nm, corresponding to the MnFe_2_O_4_ (311) crystal phase [43], confirming the successful preparation of M@BNNS. Additionally, the TEM characterization of UPy-modified BNNS is depicted in Fig. 2f. The high transparency of the nanosheet image indicates that U-BNNS retains a few-layer structure. It is worth noting that some particles appear on the surface of U-BNNS, which might be attributed to extensive and orderly aggregation of UPy molecules during the modification process due to hydrogen-bonding interactions [44]. This is also supported by the XRD curve of UPy shown in Fig. 1b. The corresponding EDS spectrum shows significant peaks for C and O elements, confirming the successful introduction of UPy on the surface of BNNS and the successful preparation of U-BNNS.

### Schematic Diagram and Microscopic Images of Different Composites

**Fig. 3:**
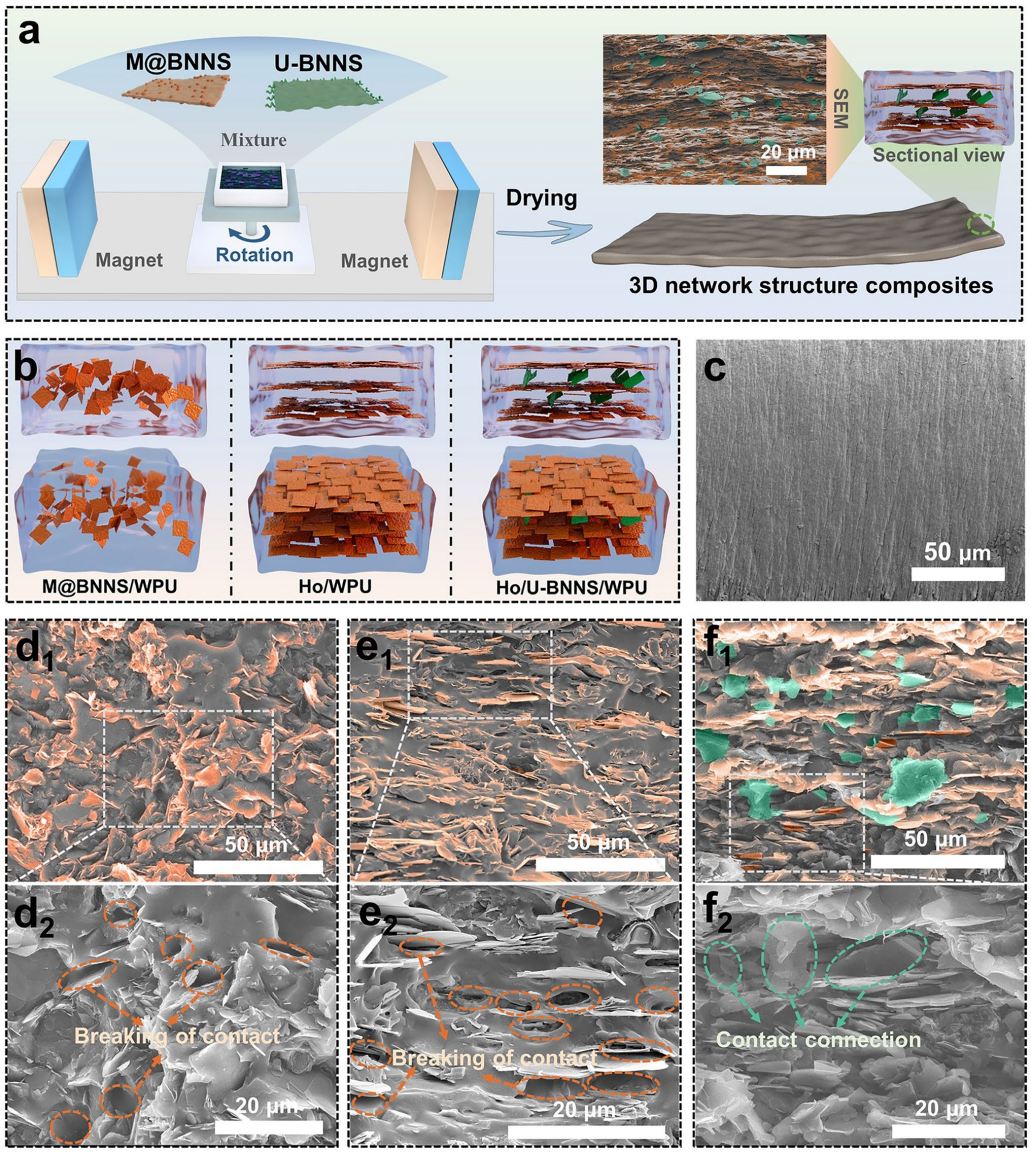
**a** Schematic diagram of the preparation of composites with 3D-bridge architecture based on magnetic field orientation; **b** schematic drawings of different structural composites; **c** SEM image of liquid nitrogen brittle cross sections of pure WPU section; SEM images of composites with different structures, **d** M@BNNS/WPU, **e** Ho/WPU, and **f** Ho/U-BNNS/WPU

The preparation process of various functional fillers and the 3D network structured composites is illustrated in Fig. 3a. A mixed polymer solution containing the WPU matrix, the magnetically responsive M@BNNS, and the non-magnetic filler U-BNNS is placed under a horizontal magnetic field for natural drying, resulting in the 3D network structured composites. Cross-sectional SEM images of the composite demonstrate that M@BNNS is well-aligned along the direction of the magnetic field, creating an ordered unidirectional continuous structure. In contrast, U-BNNS is randomly dispersed among these unidirectional structures, serving as a medium with the bridging structure that connects M@BNNS. This microstructure is through to facilitate phonon transport in the non-magnetic field direction, thereby enhancing the thermal conductivity of the composites in all directions [45]. Figure 3b presents a schematic diagram illustrating various composite structures. The M@BNNS/WPU composite is formed through simple physical mixing, resulting in a disordered structure with M@BNNS randomly dispersed within the polymer matrix. This disordered arrangement typically necessitates a high filler content (≥ 50 wt%) to establish continuous thermal conduction pathways for improved thermal conductivity [4]. In contrast, the Ho/WPU composite achieves an oriented structure by aligning the magnetically responsive M@BNNS horizontally under a magnetic field, enabling lower filler loading to create continuous pathways and enhance thermal performance in the direction of filler orientation [46]. However, the thermal conductivity in the through-plane direction remains low for the Ho/WPU composite, as noted in previous studies [33]. To address this limitation, a bridging structure is introduced between the parallel continuous pathways to form a 3D thermal conduction route. U-BNNS, which is not influenced by the magnetic field, is added to the matrix and randomly dispersed to connect the horizontal pathways formed by M@BNNS. This configuration results in the 3D-bridging structure composite, as illustrated in the Ho/U-BNNS/WPU. It is anticipated that composites featuring this bridging structure will demonstrate excellent thermal performance in both in-plane and through-plane directions.

To investigate the microstructural distribution of fillers in different composite structures, SEM is employed to examine the cross sections of the composites, as shown in Fig. 3c-f. The cross section of pure WPU appears relatively flat and smooth. In contrast, the M@BNNS/WPU composite exhibits wrinkles in its cross section, indicating that the M@BNNS fillers are randomly dispersed within the matrix, resulting in a disordered structure. The SEM image of the Ho/WPU composite as shown in Fig. 3e, reveals a regular distribution of M@BNNS along the horizontal direction, which creates an oriented structure. Higher-magnification SEM images further highlight numerous uncontacted points between the fillers and the matrix, indicating poor interaction between M@BNNS and the WPU matrix. This uneven stress distribution during the fracture of composites contributes to these disconnections. Figure 3f displays the cross section of the Ho/U-BNNS/WPU composite. Unlike the Ho/WPU composite, this structure features not only horizontally aligned M@BNNS but also incorporates randomly distributed U-BNNS, indicated by areas marked in green. These green-mark fillers bridge the horizontal pathways formed by the orange-mark fillers, resulting in a composite with 3D thermal conduction pathways. Additionally, high-magnification SEM images show satisfactory contact between the fillers and the matrix, attributed to the strong quadruple hydrogen-bonding interactions between UPy and the WPU matrix, which enhances the interaction between U-BNNS and the polymer matrix [39].

### Thermal Conductivity of Composites with Different Structures

**Fig. 4:**
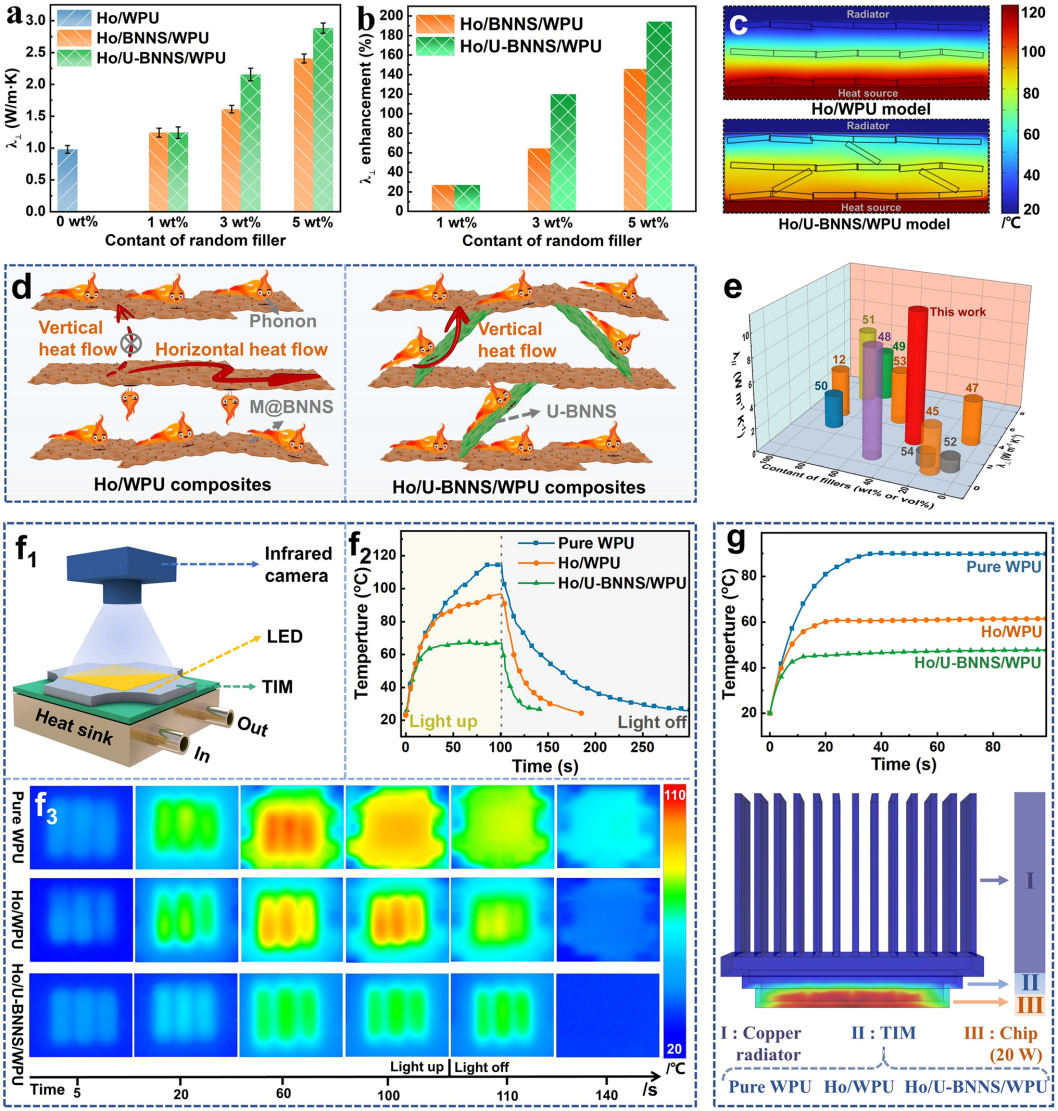
**a** Through-plane thermal conductivity of composites with different structures; **b** statistical chart of the percentage improvement in the through-plane thermal conductivity of the bridge structure composite compared to the single-direction composite; **c** finite element simulation of heat transfer capacity of composites with different structures; **d** schematic diagram of thermal conductivity mechanism of composites with different structures; **e** comparison of the thermal conductivity of the 3D-bridge architecture composite with recent thermal conductivity composites; **f** schematic diagram of the thermal conductive film for LED thermal management testing; **g** finite element simulation diagrams of different composites used as TIM for silicon-based chips, along with their corresponding chip temperature curves

To explore the thermal conductivity of different composite structures, the thermal diffusivity of the composites is measured using the laser flash method, combined with specific heat capacity (measured by differential scanning calorimetry), and density (measured using an impregnation approach), which allows for the calculation of thermal conductivity for the various composites. The results are shown in Fig. 4a, with specific calculation parameters provided in Table S3. For the horizontally oriented composites, Ho/WPU, containing 30 wt% M@BNNS, the thermal conductivity in the in-plane direction (*λ*_*//*_) is 10.77 W m^−1^ K^−1^, while the through-plane (*λ*_**⊥**_) is only 0.98 W m^−1^ K^−1^. This demonstrates significant anisotropic thermal conductivity. In the in-plane direction, well-formed thermal conduction pathways exist within the interior of Ho/WPU, but in the through-plane direction, the pathways lack direct connection, leading to much lower thermal conductivity. In contrast, as fillers unaffected by magnetic fields are incorporated, the *λ*_**⊥**_ of Ho/U-BNNS/WPU improved significantly. In particular, Ho/U-BNNS/WPU with the same filler content exhibits higher *λ*_**⊥**_ compared to composites containing unmodified BNNS (Ho/BNNS/WPU). This improvement is attributed to the stronger hydrogen-bonding interactions between the UPy and WPU molecular chains, which reduce phonon scattering and enhance heat transfer between the horizontal pathways [36]. Figure 4b shows the improvement in *λ*_**⊥**_ for the composites with 3D-bridge architecture relative to the horizontally oriented Ho/WPU composite. At relatively low filler concentrations, the *λ*_**⊥**_ improved substantially. Notably, the Ho/5-U-BNNS/WPU composite, with 5 wt% U-BNNS, achieved a *λ*_**⊥**_ of 2.88 W m^−1^ K^−1^, representing a 194.2% increase compared to Ho/WPU composites. Meanwhile, Fig. S3 presents *λ*_*//*_ of composites, which reveals that the Ho/5-U-BNNS/WPU composite maintains thermal conductivity comparable to the Ho/WPU horizontally oriented composite. These results demonstrate that incorporating U-BNNS into the composites creates continuous thermal conduction pathways, improving heat transfer in both directions and enhancing the overall thermal management properties of the 3D-bridge architecture composite.

To investigate the heat conduction capabilities of composites with varying structural designs, finite element simulations are conducted on different composite models, as illustrated in Fig. 4c. Detailed parameters for the simulation process are provided in Supporting Information S2.1. The simulation results reveal that the single-oriented Ho/WPU composite model is limited in its ability to efficiently transfer heat from the heat source to the heat sink, leading to localized heat accumulation. In contrast, the 3D-bridge architecture composite model, Ho/U-BNNS/WPU, exhibits superior heat transfer performance, effectively conducting heat from the heat source to the heat sink and ensuring efficient thermal management. Based on the microstructural and thermal conductivity analyses of the different composite structures, a conceptual diagram is proposed to illustrate the enhancement in thermal conductivity facilitated by the bridge architecture as shown in Fig. 4d. Compared to Ho/WPU, the 3D-bridge architecture composite not only features a continuous M@BNNS platform that facilitates rapid horizontal heat transfer but also incorporates U-BNNS, which is unaffected by the magnetic field, to form bridges between these heat transfer platforms. These bridges establish efficient pathways that connect the horizontal heat conduction networks, leading to a marked improvement in vertical thermal conductivity. To further emphasize the unique advantages of the 3D-bridge architecture composite in enhancing thermal performance, a comparison is made between the Ho/U-BNNS/WPU composite and recent thermal conductive composites in both in-plane and through-plant directions, as shown in Fig. 4f, with detailed data provided in Table S4 [12, 45, 47–54]. The results clearly demonstrate that the Ho/U-BNNS/WPU composite not only maintains high thermal conductivity in the in-plane direction but also offers significant improvements in through-plane heat conduction. Notably, the 3D-bridge architecture composite employs a simple one-step molding process, where the composite slurry is mixed and directly dried under a magnetic field. This approach not only simplifies the preparation process but also reduces costs, making it highly suitable for large-scale commercial applications.

To assess the practical applications of differently structured composites in electronic devices, various composites are evaluated as thermal interface material (TIM) for LED lighting, focusing on their thermal management capabilities. As illustrated in the experimental schematic in Fig. 4f, the LED light is installed on a cooled heat sink, and the composites are used as TIM between the heat sink and the LED. Meanwhile, a thermal imaging camera recorded the temperature changes of the LED over time, with the resulting temperature curve presented in Fig. 4f_2_. The results indicate that when WPU is employed as TIM, the outer temperature of LED rapidly escalates, reaching 114.7 °C after 100 s of operation, followed by a gradual decline in temperature post-shutdown. In contrast, with the Ho/WPU composite as the TIM, the surface temperature of LED initially increased swiftly for the first 35 s and then rose more gradually, stabilizing at 86.3 °C after 100 s. This demonstrates that Ho/WPU provides improved thermal management compared to pure WPU. In contrast, when the 3D-bridge architecture composite is utilized as the TIM, the surface temperature of the LED equilibrated at 69.8 °C after just 25 s of operation, maintaining stability until 100 s. Upon shutting off the LED, the surface temperature quickly returned to room temperature, showcasing superior thermal management capabilities compared to the Ho/WPU composite. Furthermore, the finite element simulations are conducted to assess the thermal management performance of pure WPU and its composites when applied as TIM for electronic chips, as presented in Fig. 4g, with detailed parameters available in Supporting Information S2.2. When pure WPU is used as the TIM, the chip’s surface temperature rises sharply, stabilizing at 89.7 °C. In contrast, the single-orientation composite, Ho/WPU, significantly improved thermal management, reducing the equilibrium temperature to 60.8 °C. Remarkably, the 3D-bridged composite, Ho/U-BNNS/WPU, achieved an even lower equilibrium temperature of 47.7 °C, corresponding to reductions of 46.8% and 21.5% compared to pure WPU and Ho/WPU, respectively. These results underscore the superior thermal management capabilities of the 3D-bridged composite. Importantly, the performance and lifetime of electronic devices are closely correlated with temperature, and a reduction of 10 °C in operating temperature can enhance lifespan by 50% [1]. Therefore, the 3D-bridge architecture composite has promising applications in thermal management materials for integrated circuits and batteries.

### Flame Retardancy of Composites with Different Structures

**Fig. 5:**
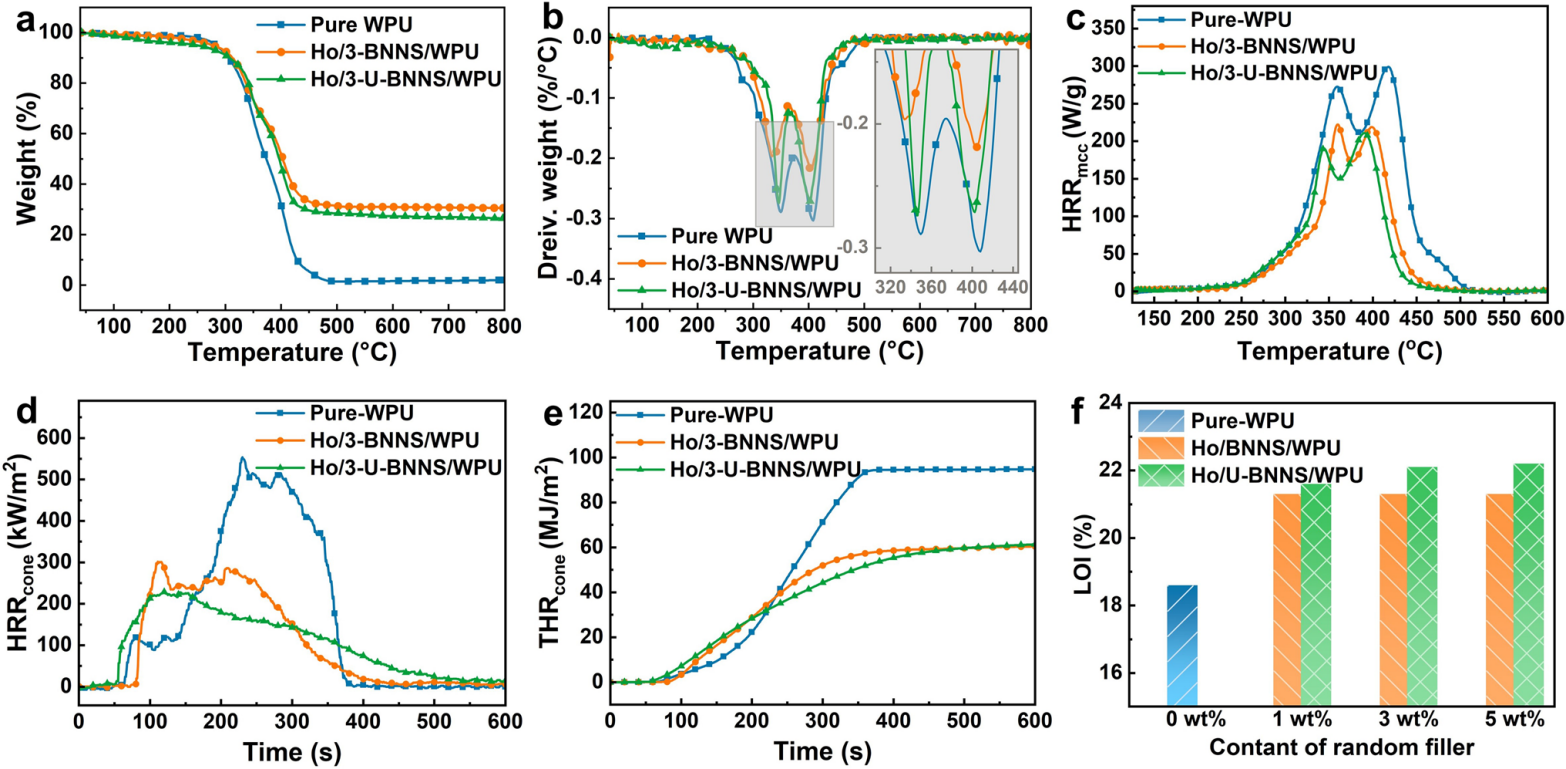
**a** Thermogravimetric curves of pure WPU and its composites, **b** the corresponding thermogravimetric differential curves. Flame-retardant performance test of pure WPU and its composites, **c** heat release rate curve according to microcalorimetric test, **d** heat release rate curve, and **e** corresponding total heat release curve according to cone calorimetric test. **f** Limiting oxygen index

Thermally conductive polymer composites employed in electronic devices are often susceptible to the risk of thermal runaway, necessitating stringent flame retardancy requirements [55]. Figure 5 presents the results of thermal stability and flame retardancy tests for various composite structures, with involved parameters as shown in Table S5. The thermogravimetry analysis (TGA) curves and corresponding differential curves in Fig. 5a, b reveal that the initial decomposition temperature (the temperature corresponding to a 5 wt% weight loss) for pure WPU is 282.1 °C, with thermal degradation occurring in two main stages. The initial thermal decomposition temperature and differential weight loss peak for the composites are lower than those for pure WPU, likely attributable to the fillers enhancing thermal conductivity, which accelerates the heating of the matrix and induces earlier decomposition. Additionally, the differential weight loss peak and char residue of the composites demonstrate significant improvements compared to pure WPU, indicating enhanced charring ability. Furthermore, as depicted in Fig. 5c, the microcalorimetry (MCC) curves for pure WPU and its composites show that thermal release primarily occurs in two stages across all materials. The peak heat release rate (*p*HRR_MCC_) for pure WPU is 298.9 W g^−1^, while the *p*HRR_MCC_ for the composites notably decreases with the addition of fillers. In particular, the Ho/U-BNNS/WPU composite exhibits a *p*HRR_MCC_ of only 210.8 W g^−1^, reflecting a reduction of 29.5% compared to pure WPU. This highlights the effective mitigation of the thermal release rate due to the incorporation of fillers in the composite.

To simulate the flame retardancy of the 3D-bridge structure composites under realistic fire conditions, cone calorimetry (CCT) tests are conducted on WPU and its composites [56], with results as shown in Fig. 5d, e. The peak heat release rate (*p*HRR_cone_) and the total heat release (THR_cone_) for WPU during combustion are recorded at 553.5 kW m^−2^ and 94.6 MJ m^−2^, respectively. The incorporation of fillers significantly reduced both *p*HRR_cone_ and THR_cone_ in the composites, particularly for the Ho/3-U-BNNS/WPU composite, which exhibited a 58.9% and 36.9% reduction in *p*HRR_cone_ and THR_cone_, respectively, compared to pure WPU, demonstrating enhanced thermal release mitigation capabilities. Moreover, smoke release during the combustion of composites is a significant fire hazard [57]. Figure S6b shows the smoke release curves for pure WPU and its composites. It is evident that the addition of functional fillers significantly reduces smoke release. Notably, the peak smoke release rate of the Ho/3-U-BNNS/WPU composite is only 0.05 m^2^ s^−1^, representing reductions of 79.2% and 37.5% compared to pure WPU and Ho/3-BNNS/WPU, respectively. Interestingly, after 350 s of combustion, the smoke release rate of Ho/3-U-BNNS/WPU gradually exceeds that of Ho/3-BNNS/WPU. This phenomenon may be attributed to the delayed combustion of the composite containing U-BNNS, resulting in continued decomposition at the later stages of the CCT test, consistent with the trends observed in the heat release rate curves. The carbon dioxide release rate curves for pure WPU and its composites exhibit a similar trend, further demonstrating that the incorporation of functional fillers effectively reduces the fire hazards associated with WPU. Notably, the ignition time for Ho/U-BNNS/WPU is significantly shorter than that of pure WPU and Ho/BNNS/WPU. This may be caused by the early decomposition of U-BNNS upon heating, which is the relatively low thermal decomposition temperature of UPy, typically ranging from 200 ~ 250 °C [58]. And, the decomposition product of UPy mixes with the combustible gases produced during WPU decomposition and diffuses into the air, leading to earlier ignition. However, the nitrogenous non-combustible gas released from the initial decomposition of U-BNNS effectively reduced heat release during the combustion of Ho/3-U-BNNS/WPU, showcasing an efficient gas-phase flame-retardant mechanism [59].

Additionally, the limiting oxygen index (LOI) comprehensively evaluated the flammability of pure WPU and its composites, as illustrated in Fig. 5f. The LOI value of pure WPU is low, only 18.6%. In contrast, the LOI values of the composites increased significantly with the addition of fillers. Notably, composites containing unmodified BNNS showed little change in LOI values across varying amounts, remaining at 21.6%. In contrast, the LOI values of Ho/U-BNNS/WPU composites further increased with the addition of U-BNNS. This improvement is likely due to the relatively simple flame retardancy mechanism of composites with only BNNS, which relies primarily on the barrier effect of the 2D material. In contrast, the UPy-modified filler, by releasing nitrogenous non-combustible gases during decomposition, dilutes combustible gases and reduces oxygen concentration, leading to a synergistic effect with the barrier properties of the two-dimensional materials [57], which demonstrates superior flame retardancy.

**Fig. 6:**
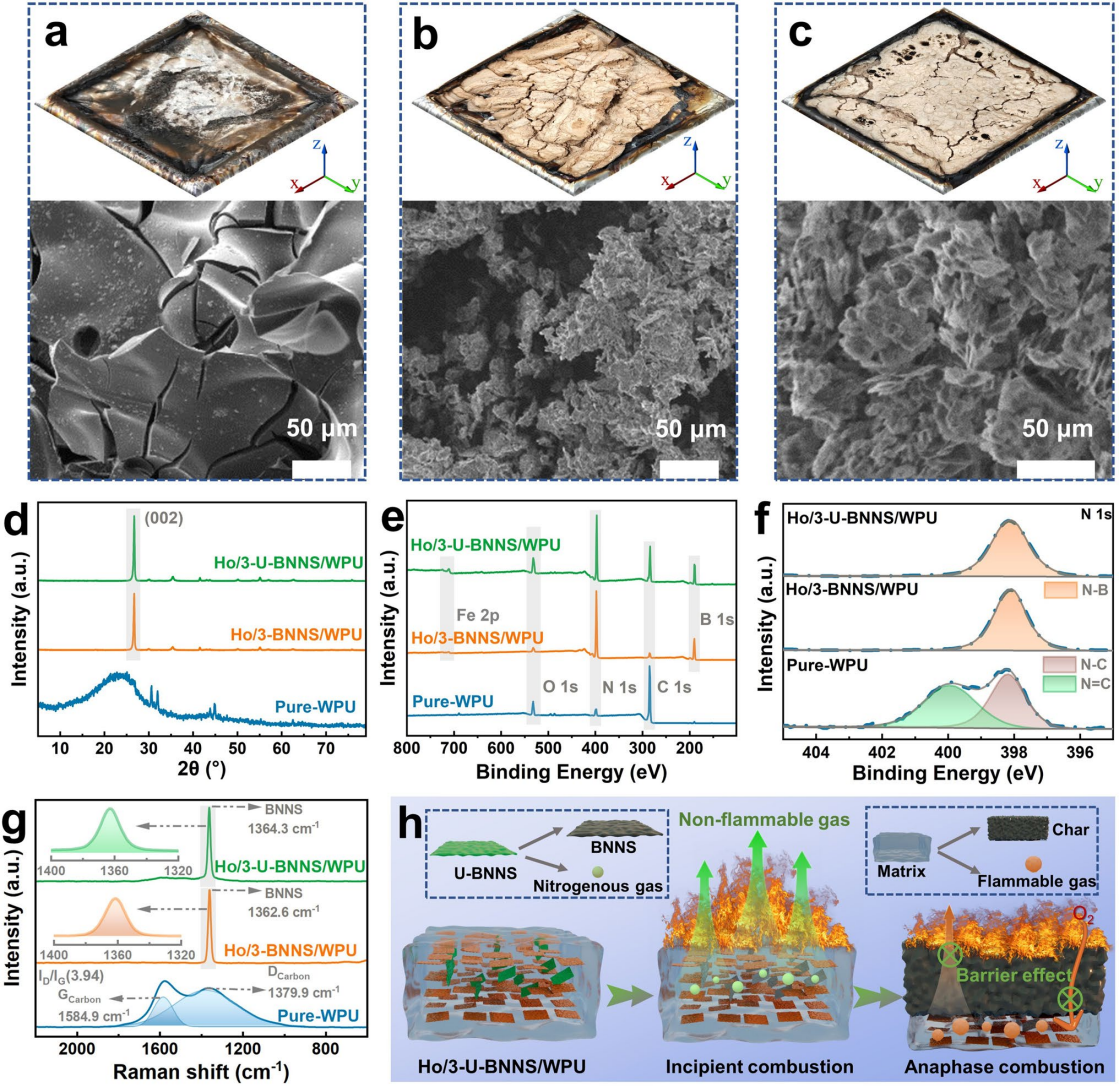
Digital photographs and the corresponding SEM images for the char residue of **a** WPU, **b** Ho/BNNS/WPU, and **c** Ho/U-BNNS/WPU after combustion; the surface structure and elemental composition of pure WPU and its composites after combustion are characterized by **d** XRD, **e** XPS survey spectrum, **f** N 1*s* XPS fine spectrum and **g** Raman spectrum; **h** schematic diagram of the flame-retardant mechanism of the 3D-bridge structure composite

To further elucidate the flame retardancy mechanism of the 3D-bridge structure composites, the residual char morphology, elemental composition, and structure of pure WPU and its composites after cone calorimetry tests are characterized, as shown in Fig. 6. Photographs and corresponding micrographs of the residual char in Figs. 6a-c and S6a which demonstrate that pure WPU produces minimal residual char upon combustion, whereas composites containing fillers exhibit significant amounts of yellow–brown residual char. Notably, the micrographs reveal that the residual char from the U-BNNS composites appears continuous and dense char layer compared to that from pure WPU and Ho/BNNS/WPU composites. Figure S6b presents the mass loss curves during combustion, providing a more intuitive demonstration of the results. Notably, pure WPU exhibits almost no residual char after combustion, with a final mass of only 0.7 g, which is significantly lower than that of the composites containing functional fillers, such as Ho/3-BNNS/WPU (13.1 g) and Ho/3-U-BNNS/WPU (13.2 g). This dense and cohesive char layer acts as a protective layer during combustion, resisting the decomposition diffusion from the matrix to the combustion layer and slowing the diffusion of oxygen in air to matrix, thereby reducing thermal release and enhancing flame retardancy [60]. Figure 6d-g presents the elemental composition and structural characterization of the residual char. The XRD patterns indicate that the residual char of the composites after combustion exhibits a characteristic peak (002) corresponding to BNNS [47]. Importantly, all UPy-related characteristic peaks observed in Fig. 1b are absent in the residual char, indicating the complete decomposition of UPy during combustion, consistent with the gas-phase flame retardancy mechanism discussed in Fig. 5a. Additionally, the XPS total spectra of the char reveal that pure WPU mainly consists of C, N, and O elements, while the residue char after combustion of composites contains these elements alongside characteristic peaks for Mn 2*p* and Fe 2*p*, which explains the yellow–brown coloration of the residual char. High-resolution spectral analysis of N 1*s* in the residual char shows that the char from the filler-containing composites exhibits a characteristic peak at 389.4 eV, corresponding to N-B [22], confirming the presence of BNNS or its derivatives. The Raman spectra of residual char for pure WPU and its composites are shown in Fig. 6g; it is evident that the graphitization degree of pure WPU residual char is low, with an I_D_/I_G_ ratio of 3.94. In contrast, the residual char of composites with functional fillers exhibits extremely strong characteristic peaks of BNNS [61], indicating that the structure of BNNS remains largely intact after combustion. The primary component of the residual char is BNNS, a result consistent with the XPS and XRD analysis of the residual char. Figure 6h illustrates the proposed flame retardancy mechanism during the combustion of the bridge structure composites. The combustion process can be divided into two distinct stages. In the initial stage, the decomposition products of U-BNNS release nitrogen-containing non-combustible gases, which dilute both the flammable gases emitted from the substrate and the surrounding oxygen. This dilution reduces early heat release during combustion, demonstrating an effective gas-phase flame-retardant mechanism [59, 62]. In the second stage, the two-dimensional BNNS materials generated from U-BNNS decomposition, together with the horizontally oriented M@BNNS filler, form robust physical barriers. These barriers effectively restrict the diffusion of flammable gases and oxygen between the substrate and the combustion layer, thereby reducing heat release, delaying combustion, and exhibiting a condensed-phase flame-retardant mechanism. Therefore, the 3D-bridge structure composites effectively integrate gas-phase and condensed-phase flame retardancy mechanisms, enhancing fire safety and reducing the risks of thermal runaway in thermal conductive composite applications, thereby offering valuable insights for the design of advanced heat management materials.

## Conclusions

In summary, this work develops a composite with 3D network continuous pathway, Ho/U-BNNS/WPU, by simultaneously incorporating magnetically responsive nanosheet (M@BNNS) and non-magnetic nanosheet (U-BNNS) into a WPU matrix to synchronous molding under a horizontal magnetic field. The 3D-bridge structure is created using a one-step molding method, where M@BNNS forms the in-plane heat conduction path aligned under a horizontal magnetic field, combined with the bridging structure established by U-BNNS, which effectively establishes a three-dimensional continuous pathway within Ho/U-BNNS/WPU. This microstructural design enabled the composite to exhibit exceptional in-plane (*λ*_***//***_) and through-plane thermal conductivities (*λ*_**⊥**_). In particular, with the addition of 30 wt% M@BNNS and 5 wt% U-BNNS, the *λ*_***//***_ and *λ*_**⊥**_ of composites reach 11.47 and 2.88 W m^−1^ K^−1^, respectively, which representing a 194.2% improvement in *λ*_**⊥**_ compared to composites with a single orientation of M@BNNS. Finite element simulations confirmed the exceptional thermal conduction capabilities of Ho/U-BNNS/WPU. Moreover, the composite exhibited remarkable flame retardancy, with reductions of 58.9% in peak heat release rate and 36.9% in total heat release compared to pure WPU. The analysis of the residual char morphology and elemental composition reveals a synergistic gas-phase and condensed-phase flame-retardant mechanism. Additionally, Ho/U-BNNS/WPU demonstrated superior practical thermal management performance when employed as a thermal interface material for LED and chip cooling. Thus, this work not only presents a novel approach to the structural design and efficient flame retardant of thermally conductive composites but also offers promising applications in flame-retardant thermal management materials for integrated circuits and batteries.

## Supplementary Information

Below is the link to the electronic supplementary material.

Supplementary file1 (DOCX 2938 KB)

Supplementary file2 (MP4 8184 KB)
