# Supplementary material for: Highly Thermally Conductive and Flame-Retardant Waterborne Polyurethane Composites with 3D BNNS Bridging Structures via Magnetic Field Assistance
Source: Nanomicro Lett. 2025 Feb 7;17:138. doi: 10.1007/s40820-025-01651-1 (PMC11805750; doi:10.1007/s40820-025-01651-1)
Supplement: Supplementary file 1 — Supplementary file1 (DOCX 2938 KB) [file 40820_2025_1651_MOESM1_ESM.docx]

Supporting Information for

**Highly Thermally Conductive and Flame-retardant Waterborne Polyurethane Composites With 3D BNNS bridging Structures *via* Magnetic Field Assistance**

Hao Jiang^1, 2^, Yuhui Xie^1^, Mukun He^2^, Jindao Li^1^, Feng Wu^1^, Hua Guo^2^, Yongqiang Guo^2^, Delong Xie^1,^ *, Yi Mei^1^, Junwei Gu^2,^ *

^1^ Yunnan Provincial Key Laboratory of Energy Saving in Phosphorus Chemical Engineering and New Phosphorus Materials, The International Joint Laboratory for Sustainable Polymers of Yunnan Province, The Higher Educational Key Laboratory for Phosphorus Chemical Engineering of Yunnan Province, Faculty of Chemical Engineering, Kunming University of Science and Technology, Kunming, Yunnan 650500, P. R. China

^2^ Shaanxi Key Laboratory of Macromolecular Science and Technology, School of Chemistry and Chemical Engineering, Northwestern Polytechnical University, Xi’an, Shaanxi 710072, P. R. China

*Corresponding authors. E-mail: [cedlxie@kust.edu.cn](mailto:cedlxie@kust.edu.cn) (Delong Xie); [gjw@nwpu.edu.cn](mailto:gjw@nwpu.edu.cn) (Junwei Gu)

**S1 Experimental Section**

The exfoliation of *h*-BN and magnetic modification of boron nitride nanosheets (M@BNNS) can refer to our previous work [S1, S2]. In specifically, the h-BN power undergoes a mixed alkali aqueous hydrothermal treatment and high-speed shearing to exfoliate into BNNS. And, the hybridization of BNNS and MnFe_2_O_4_ involved the in-situ synthesis of MnFe_2_O_4_ on the surface of BNNS. And, the mass ratio of BNNS to MnFe_2_O_4_ in the synthesized M@BNNS was deliberately set at 10:1.

**S1.1 Preparation of UPy**

6-Methylisocytosine (MIC) was subjected to vacuum drying and dehydration treatment one day before the synthesis reaction. A total of 0.05 mol of MIC was placed in a three-neck flask connected to a double-manifold. Subsequently, 0.7 mol of hexamethylene diisocyanate (HDI) was added, and the mixture was stirred thoroughly to ensure uniformity. The system was then evacuated using the double-manifold and purged with nitrogen gas three times to maintain an inert atmosphere. The reaction temperature was set at 90°C, and the reaction was allowed to proceed for 12 h. Upon completion, the mixture was subjected to rotary evaporation to yield a white solid. The crude product was washed three times with a hexane solution via vacuum filtration to remove excess HDI. Finally, the solid was transferred to a vacuum oven and dried at 60°C for 24 h to obtain the final product, UPy.

**S1.2 Preparation of U-BNNS**

U-BNNS was synthesized through the reaction between the isocyanate groups at one end of UPy and the hydroxyl groups on the surface of BNNS. Specifically, 0.2 g of UPy powder was placed in a three-neck flask, followed by the addition of 20 mL of dimethylformamide (DMF). The mixture was stirred and sonicated for 30 min under a nitrogen atmosphere to ensure the complete dissolution of UPy. Subsequently, 2 g of BNNS was dispersed in 30 mL of DMF, which was also stirred and sonicated for 30 min under nitrogen to achieve uniform dispersion. The BNNS dispersion was then transferred into the three-neck flask containing the UPy solution. The flask was placed in an oil bath, and magnetic stirring was initiated while connecting it to a double manifold. To the mixture, 0.2 mL of dibutyltin dilaurate was added, and the reaction system was evacuated and purged with nitrogen three times, followed by continuous nitrogen bubbling to maintain a positive pressure atmosphere. The oil bath was then heated to 80°C and maintained at that temperature for 8 h. After the reaction, the oil bath was allowed to cool, and the mixture was centrifuged at 12000 rpm with deionized water for three washes to collect the white precipitate. The precipitate was subsequently transferred to a vacuum oven and dried at 60°C for 24 h to obtain the final product, U-BNNS.

**S1.3** **Characterization**

Morphology and the corresponding EDS analyses were conducted using a scanning electron microscope (SEM Hitachi SU8010, Japan) at an accelerating voltage of 15 kV. Transmission electron microscopy (TEM) was performed on Tecnai G2 F20 S-TWIN (Thermo Scientific, USA) with a 200 kV accelerating voltage. Atomic force microscopy (AFM) was conducted on a DI Multimode V scanning probe microscope (Veeco Co., Ltd., USA) in the tapping mode. Fourier transform infrared (FTIR) spectra were obtained on a spectrometer (Bruker, Tensor-II) was used to obtain FTIR spectra. The X-ray diffraction (XRD) patterns of the samples and residues were conducted using an X-ray diffractometer (PAN alytical, X’ Pert-3, Netherlands) with Cu Kα radiation (λ = 0.15406 nm). A continuous scan mode with a step degree of 0.02° and a scan speed of 2.0° min^−1^ was utilized in the range from 5° to 90°. X-ray photoelectron spectroscopy (XPS) was conducted using an XSAM800 (Thermo Scientific, USA) with the Al Kα radiation (hv =1486.6 eV). The thermal behavior of all samples was evaluated via Thermogravimetry Analysis (TGA) performed on a STA449F3 thermogravimeter (NETZSCH, Germany) under nitrogen from 40 to 800 ℃ under nitrogen at the heating rate of 20 ℃·min^-1^. The mechanical property of samples was investigated on a mechanical property test instrument (Vance, ETM104B) with a gauge length of 50 mm and a loading rate of 2 mm/min. Each sample was tested five times, and the averaged data were reported. The system for LED thermal management testing was composed of a 30W LED lamp, water-cooled heat sinks, and an infrared camera for capturing the surface temperature evolutions with time. The thermal conductivity of pure WPU was measured by a heat source method (Hot Disk TPS 2500 S, Hot Disk AB, Switzerland). The through-thickness thermal conductivity (*λ*_⊥_) of composite films was calculated based on the equation *λ***_⊥_** = α × ρ × c_p_, where thermal diffusivity (α) was measured by a laser flash apparatus (LFA 467 Nanoflash, NETZSCH, Germany), The density (ρ) was measured by Archimedes method in silicone oil, and the heat capacity (c_p_) was used to measure at a heat ingrate of 10 °C min^−1^ by differential scanning calorimetry (DSC3, Mettler Toledo, Switzerland).

**Table S1** Content of component of composites

| **Sample No.** | **WPU (g)** | **M@BNNS (g)** | **BNNS (g)** | **U-BNNS (g)** |
| --- | --- | --- | --- | --- |
| WPU | 10 | 0 | 0 | 0 |
| Ho/WPU | 7 | 3 | 0 | 0 |
| Ho/1-BNNS/WPU | 6.9 | 3 | 0.1 | 0 |
| Ho/3-BNNS/WPU | 6.7 | 3 | 0.3 | 0 |
| Ho/5-BNNS/WPU | 6.5 | 3 | 0.5 | 0 |
| Ho/1-U-BNNS/WPU | 6.9 | 3 | 0 | 0.1 |
| Ho/3-U-BNNS/WPU | 6.7 | 3 | 0 | 0.3 |
| Ho/5-U-BNNS/WPU | 6.5 | 3 | 0 | 0.5 |


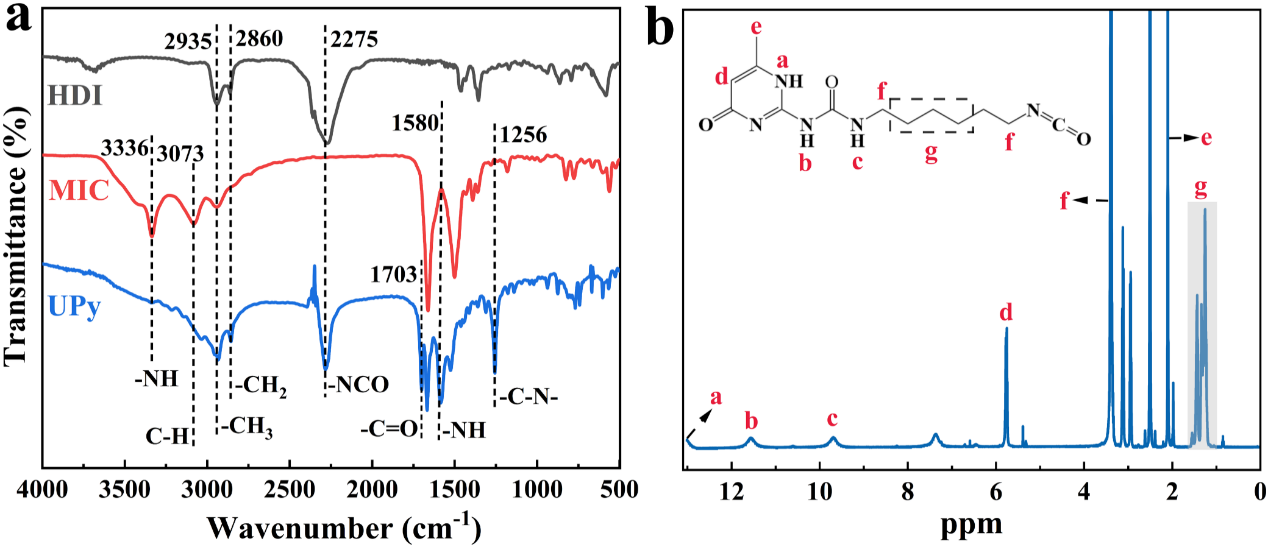


**Fig. S1** (**a**) FTIR spectra of HDI, MIC, and UPy; (**b**) ^1^H NMR spectrum of UPy


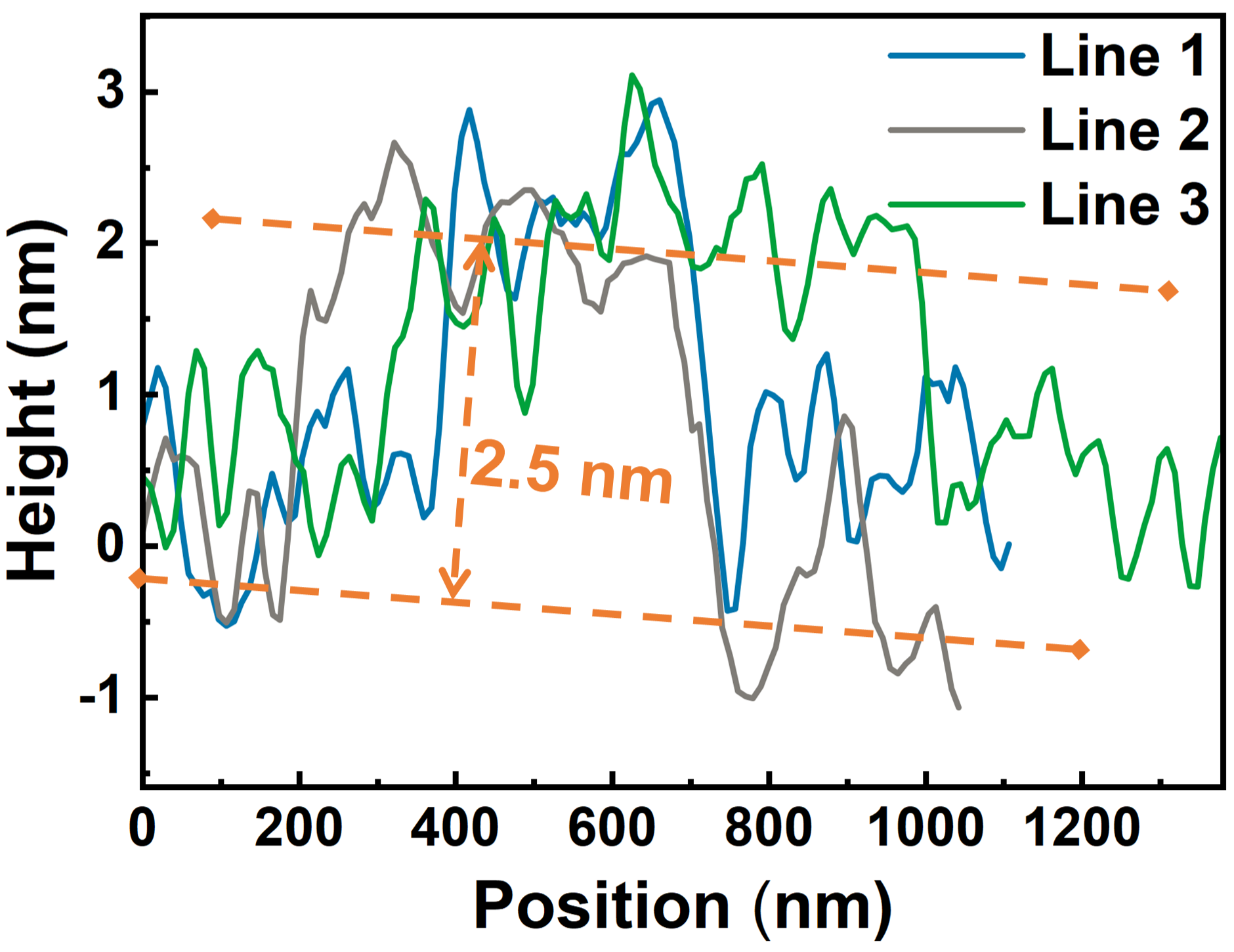


**Fig. S2** AFM image of BNNS identifies the height of each line segment


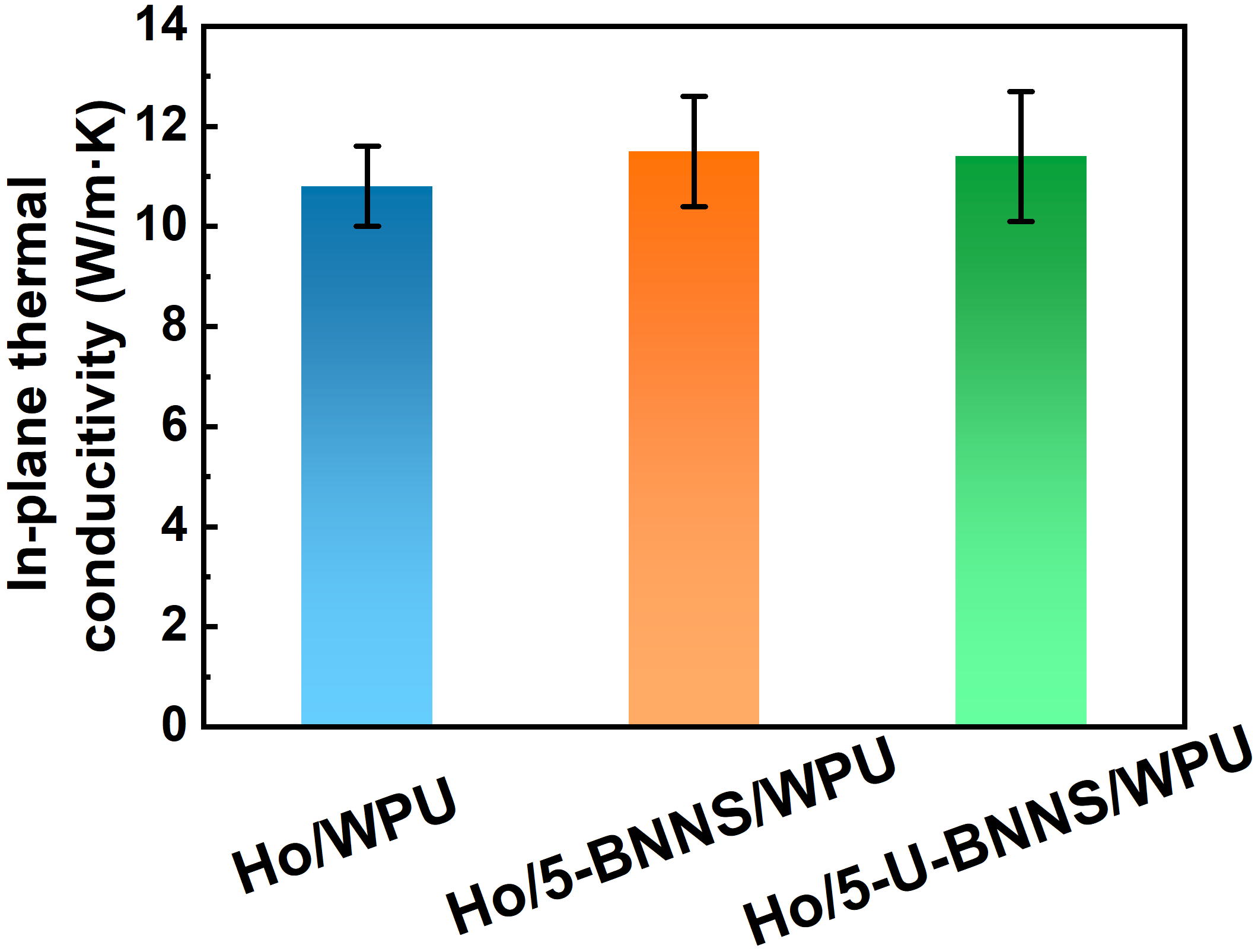


**Fig. S3** In-plane thermal conductivity of composites with different structures

**S2 Finite Element Simulation**

**S2.1 Heat transfer capacity of composites with different structures**


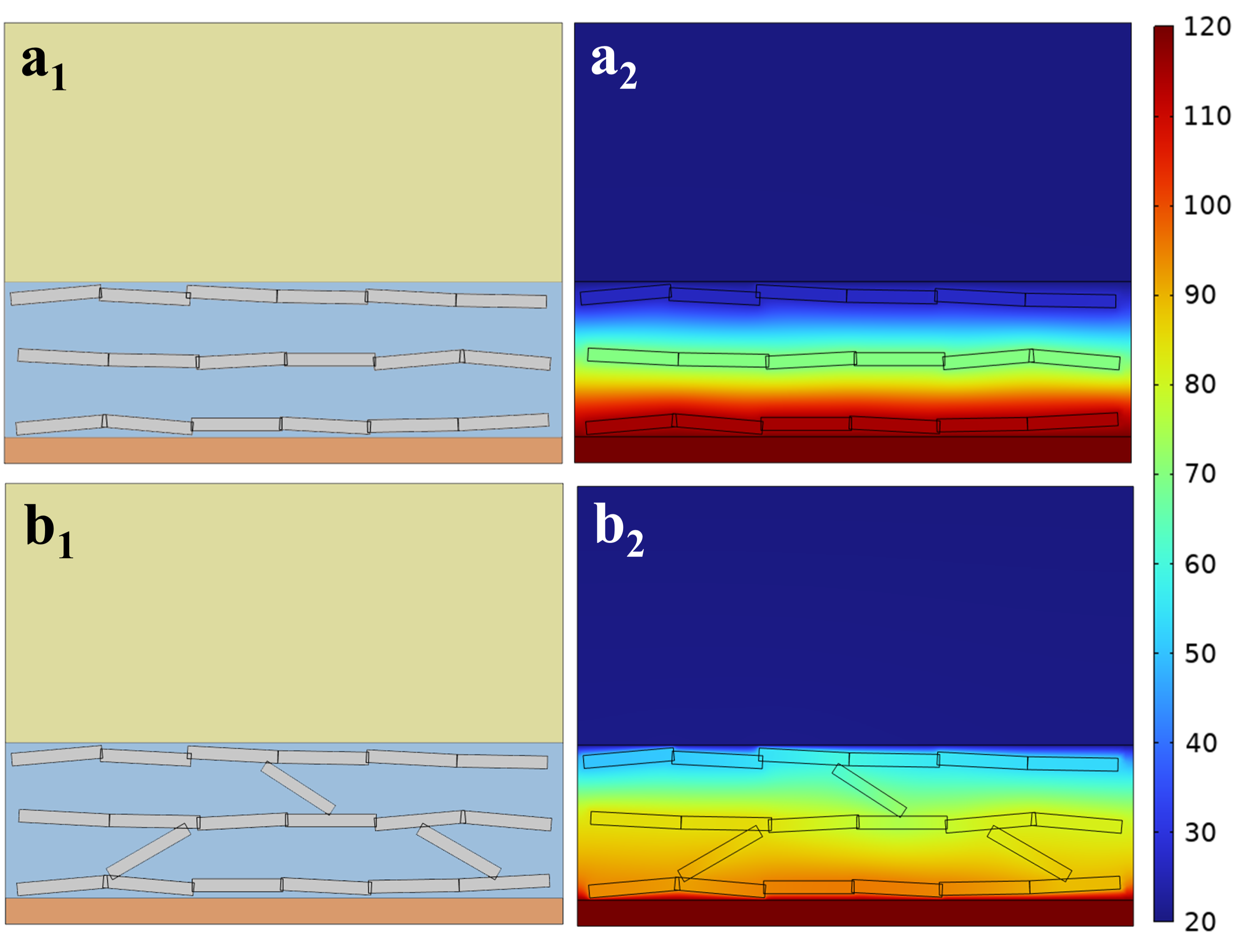


**Fig. S4** The models of the (**a**) Ho/WPU and (**b**) Ho/U-BNNS/WPU used in the COMSOL simulation

To evaluate the thermal conduction performance of composites with different structures, finite element simulations were performed using COMSOL Multiphysics on both the single-oriented Ho/WPU composite and the 3D-bridge structure composite Ho/U-BNNS/WPU. In the typical solid heat conduction model, a constant heat source of 120 °C was applied to the bottom of the composite, while a copper heat sink was placed at the top with its temperature set to 20 °C. The remaining boundaries were defined as thermal insulators and heat transfer was modeled as contact-based solid conduction. Based on literature and experimental data [S3], the thermal conductivities of filler and WPU were set at 300 W/m·K and 0.245 W/m·K, respectively. As shown in **Fig. S4**, the simulated temperature distributions of the Ho/WPU and Ho/U-BNNS/WPU composites reveal that the top surface temperature of the Ho/U-BNNS/WPU composite was higher than that of the Ho/WPU, indicating superior heat conduction. This enhancement is attributed to the limited vertical connectivity between layers in the unidirectionally aligned Ho/WPU composite, which impedes heat transfer. In contrast, the 3D-bridge structure composite facilitates vertical heat conduction through its interlayer bridges formed by distributed filler, resulting in significantly improved overall thermal performance.

**S2.2 Diagrams of different composites used as TIM for silicon-based chips**


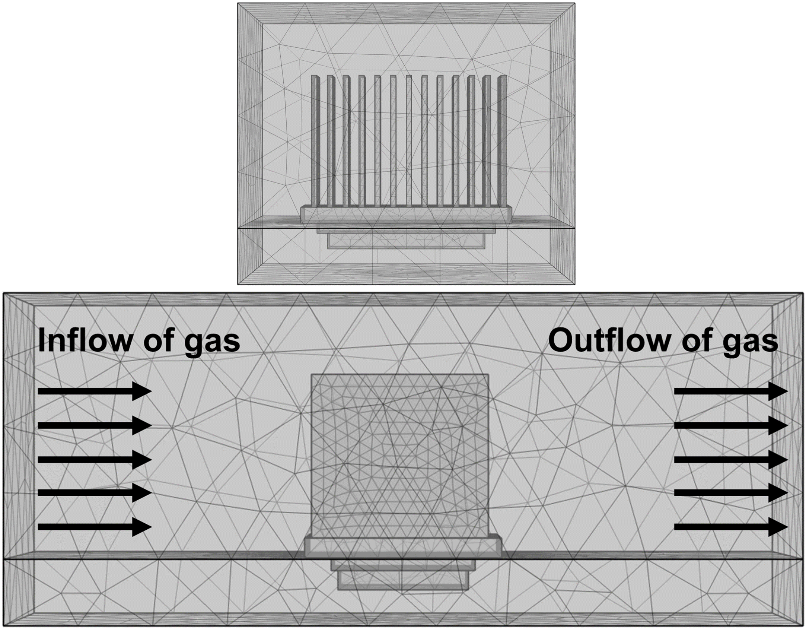


**Fig. S5** Finite element simulation model of composites as TIM in silicon chip thermal management applications

The multiphysics simulation software COMSOL was employed to simulate the thermal management efficiency of various films in cooling silicon chips. In a typical solid-fluid heat transfer finite element model, depicted in Fig. S5, the system comprises a silicon-based chip, interface filling material (TIM), and heat sink immersed in natural air convection. The heat generated by the silicon-based chip is conducted through the TIM to the heat sink and subsequently dissipated into the air. Specifically, a silicon-based chip (20 W) acts as the heat source, utilizing a copper heat sink, while air velocity is maintained at 1.0 m/s. Initial temperatures are uniformly set to 20 °C, and the TIM thickness is con**Fig.**d to be one-quarter of the silicon-based chip thickness. The materials considered include pure WPU, Ho/WPU, and Ho/U-BNNS/WPU, with experimentally determined thermal conductivity values.

**
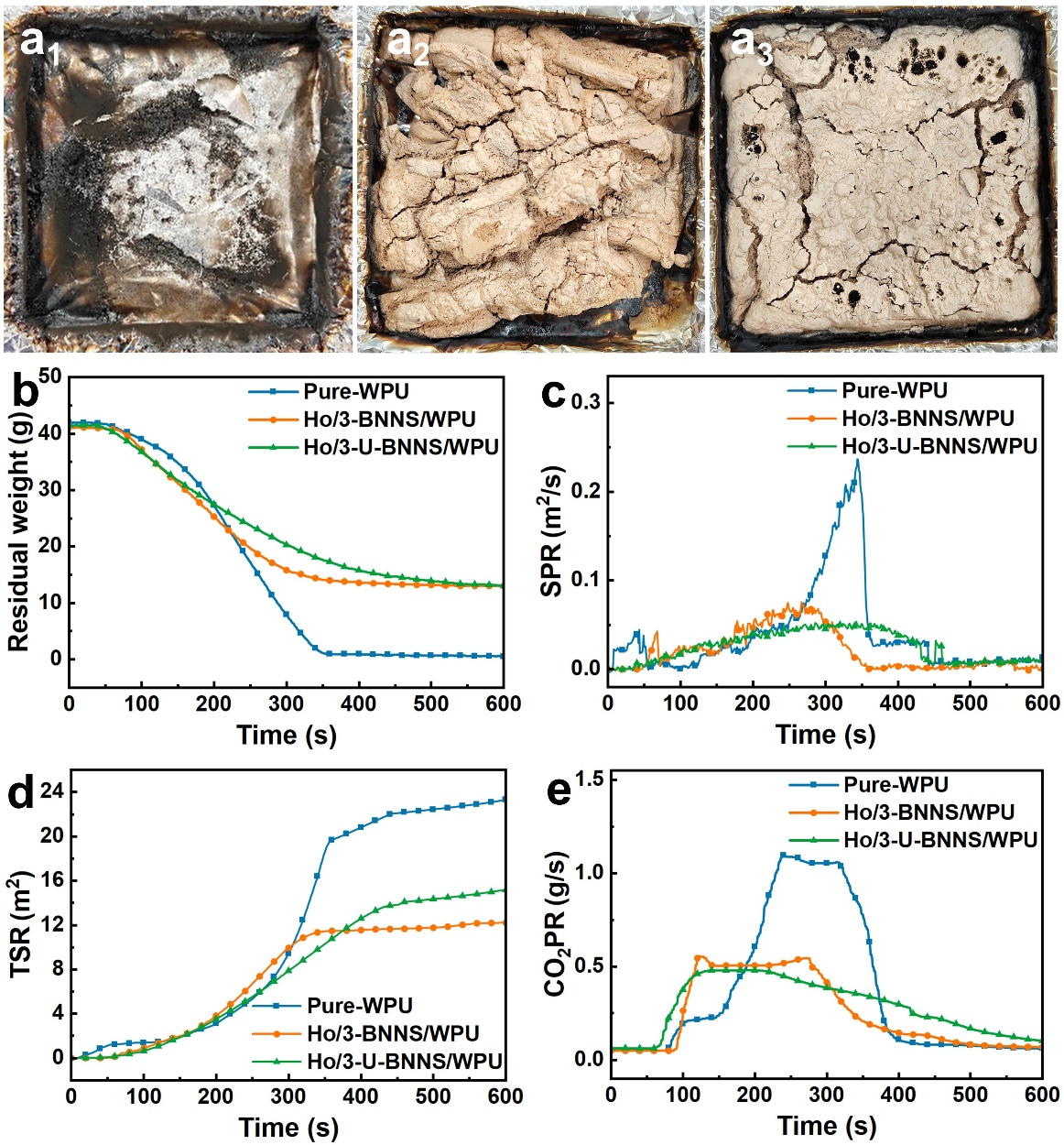
**

**Fig. S6** Digital photos of residual char of (**a_1_**) pure WPU, (**a_2_**) Ho/3-BNNS/WPU, and (**a_3_**) Ho/3-U-BNNS/WPU; (**b**) The mass loss curves, (**c**) SPR curves, (**d**) TSR curves, and (**e**) CO_2_PR curve of pure WPU and its composites obtained by cone calorimetry

**Table S2** Detail of XPS high-resolution spectrum peaking of fillers

| Spectrum | Sample | Peak position (eV) | Description |
| --- | --- | --- | --- |
| N 1s | BNNS | 397.98 | N-B |
|  | UPy | 398.78 | N=C(O) |
|  |  | 400.18 | N-C(O) |
|  | U-BNNS | 398.08 | N-B |
|  |  | 400.18 | N-C(O) |
| Fe 2p | MnFe_2_O_4_ | 711.18 | Fe 2p_1/2_ of the Fe^2+^ |
|  |  | 713.78 | Fe 2p_1/2_ of the Fe3^+^ |
|  |  | 719.08 | Satellite peak |
|  |  | 724.38 | Fe 2p_3/2_ of the Fe^2+^ |
|  |  | 727.48 | Fe 2p_3/2_ of the Fe^3+^ |
|  |  | 733.08 | Satellite peak |
|  | M@BNNS | 711.18 | Fe 2p_1/2_ of the Fe^2+^ |
|  |  | 713.88 | Fe 2p_1/2_ of the Fe3^+^ |
|  |  | 719.08 | Satellite peak |
|  |  | 723.88 | Fe 2p_3/2_ of the Fe^2+^ |
|  |  | 726.78 | Fe 2p_3/2_ of the Fe^3+^ |
|  |  | 732.38 | Satellite peak |

**Table S3** The parameters for the calculation of thermal conductivity of the composite films

| Samples No. | Through-plane thermal diffusivity (mm^2^ s^-1^) | In-plane Thermal diffusivity  (mm^2^ s^-1^) | Density (g·cm^-3^) | Specific heat capacity  (J·g^-1^ K^-1^) |
| --- | --- | --- | --- | --- |
| Ho/WPU | 0.394 | 4.351 | 1.25 | 1.98 |
| Ho/1-BNNS/WPU | 0.497 | / | 1.27 | 1.97 |
| Ho/3-BNNS/WPU | 0.634 | / | 1.31 | 1.94 |
| Ho/5-BNNS/WPU | 0.924 | 4.422 | 1.35 | 1.93 |
| Ho/1-U-BNNS/WPU | 0.506 | / | 1.26 | 1.95 |
| Ho/3-U-BNNS/WPU | 0.861 | / | 1.29 | 1.94 |
| Ho/5-U-BNNS/WPU | 1.135 | 4.518 | 1.33 | 1.91 |

**Table S4** The parameters for comparison of the thermal conductivity of the bridge architecture composite with the recent thermal conductivity composite

| Filler type and content | Matrix | Method | *λ_//_* (W·m^-1^ K^-1^) | *λ***_⊥_**(W·m^-1^ K^-1^) | Refs. |
| --- | --- | --- | --- | --- | --- |
| LC-CNT /15 wt% | LC-PI | TPS | 4.02 | 0.55 | [S4] |
| MgO@Ni@CNT /5 wt% | Silicone | TPS | 3.94 | 3.94 | [S5] |
| LM–VGA /75.9 wt% | BPDMS | LFA | 4.47 | 7.11 | [S6] |
| Al_2_O_3_/BN /86.6 wt% | Silicone | TPS | 2.9 | 2.9 | [S7] |
| β-Si_3_N_4_ /53 vol% | EP | TPS | 4.7 | 4.7 | [S8] |
| AgNWs@BNNS /50 wt% | ANF | TPS | 9.44 | 0.75 | [S9] |
| BNNS@AgNPs /20 wt% | EP | LFA | 1.13 | 1.13 | [S10] |
| AlN /90 wt% | Polydibutene | SSM | 4.21 | 4.21 | [S11] |
| AlN/BN /85 wt% | Silicone | LFA | 6.56 | 6.56 | [S12] |
| BNNS@MS /6 wt% | PVA | TPS | 1.12 | 1.12 | [S13] |
| M@BNNS/ 30 wt%  U-BNNS/ 5 wt% | WPU | LFA | 11.47 | 2.88 | This work |

1. In the test methods, LFA for the laser flash method, TPS for the transient plane heat source method, and SSM for the standard steady-state method.

**Table S5** TGA, MCC, and CCT results for WPU and its composites

| Sample No. | Pure WPU | Ho/3-BNNS/WPU | Ho/3-U-BNNS/WPU |
| --- | --- | --- | --- |
| *T*_5%_ (°C) | 282.1 | 276.6 | 249.4 |
| *T*_max-1_ (°C) | 349.5 | 333.8 | 346.1 |
| *R* _max-1_ (%/°C) | -0.29 | -0.19 | -0.27 |
| *T*_max-2_ (°C) | 407.8 | 403.6 | 402.5 |
| *R* _max-2_ (%/°C) | -0.30 | -0.22 | -0.27 |
| *Y* (wt%) | 1.7 | 30.6 | 26.8 |
| *p*HRR_mcc_ (W/g) | 298.9 | 222.2 | 210.8 |
| TTI (s) | 63 | 76 | 52 |
| Residual weight (g) | 0.7 | 13.1 | 13.2 |
| *p*CO_2_PR (g/s) | 1.10 | 0.55 | 0.48 |
| *p*SPR (m^2^/s) | 0.24 | 0.08 | 0.05 |
| TSR (m^2^) | 23.2 | 11.7 | 15.1 |
| *p*HRR_cone_ (kW/m^2^) | 553.5 | 301.3 | 227.4 |
| THR_cone_ (MJ/g) | 94.6 | 59.0 | 59.7 |

1. *T*_5%_ and *T*_max_ refer to the temperatures where at 5 wt% weight loss and the maximum weight loss rate occur, respectively;
2. *R* _max_ refers to the maximum weight loss rate; *Y* refers to the Char residues at 800 °C;
3. *p*HRR refers to the peak heat release rate; THR refers to the total heat release.
4. *p*CO_2_PR and SPR refers to the peak carbon dioxide release rate and the peak smoke release rate of composites, respectively; TSR refers to the total smoke release rate of composites;
5. TTI refers to the time to ignition of composites during Cone test.

**Supplementary References**

1. H. Jiang, J. Li, Y. Xie, Y. Du, J. Zhao et al., Rapid exfoliation and surface hydroxylation of high-quality boron nitride nanosheets enabling waterborne polyurethane with high thermal conductivity and flame retardancy. Adv. Compos. Hybrid Mater. **7**(1), 8 (2024). <https://doi.org/10.1007/s42114-023-00818-x>
2. H. Jiang, J. Li, Y. Xie, H. Guo, M. He et al., Design of efficient microstructured path by magnetic orientation boron nitride nanosheets/MnFe_2_O_4_ enabling waterborne polyurethane with high thermal conductivity and flame retardancy. J. Mater. Sci. Technol. **209**, 207-218 (2025). <https://doi.org/10.1016/j.jmst.2024.05.013>
3. Q. Yan, W. Dai, J. Gao, X. Tan, L. Lv et al., Ultrahigh-aspect-ratio boron nitride nanosheets leading to superhigh in-plane thermal conductivity of foldable heat spreader. ACS Nano **15**(4), 6489-6498 (2021). <https://doi.org/10.1021/acsnano.0c09229>
4. K. Ruan, X. Shi, Y. Zhang, Y. Guo, X. Zhong et al., Electric-field-induced alignment of functionalized carbon nanotubes inside thermally conductive liquid crystalline polyimide composite films. Angew. Chem. Int. Ed. **62**(38), e202309010 (2023). <https://doi.org/https://doi.org/10.1002/anie.202309010>
5. F. You, Y. Chen, Y. Shen, Y. Ke, G. Tong et al., Constructing a 3D interlinked network with magnetic/dielectric loss and electron/phonon co-transfer in Mg@Ni@C and Mg@Ni@CNT foams for low loads and prominent thermal/electromagnetic performance. Chem. Eng. J. **480**, 147975 (2024). <https://doi.org/10.1016/j.cej.2023.147975>
6. Q. He, M. Qin, H. Zhang, J. Yue, L. Peng et al., Patterned liquid metal embedded in brush-shaped polymers for dynamic thermal management. Mater. Horizons. **11**(2), 531-544 (2024). <https://doi.org/10.1039/D3MH01498C>
7. Z. Lin, H. Jin, H. Deng, Z. Zu, H. Huang et al., Robust, self-healable, recyclable and thermally conductive silicone composite as intelligent thermal interface material. Compos. Struct. **332**, 117932 (2024). <https://doi.org/10.1016/j.compstruct.2024.117932>
8. A. Shimamura, Y. Hotta, H. Hyuga, M. Hotta, K. Hirao, Improving the thermal conductivity of epoxy composites using a combustion-synthesized aggregated β-Si_3_N_4_ filler with randomly oriented grains. Sci. Rep. **10**(1), 14926 (2020). <https://doi.org/10.1038/s41598-020-71745-w>
9. Y. Han, K. Ruan, J. Gu, Multifunctional thermally conductive composite films based on fungal tree-like heterostructured silver nanowires@boron nitride nanosheets and aramid nanofibers. Angew. Chem. Int. Ed. **62**(5), e202216093 (2023). <https://doi.org/10.1002/anie.202216093>
10. C. Chen, Y. Xue, Z. Li, Y. Wen, X. Li et al., Construction of 3d boron nitride nanosheets/silver networks in epoxy-based composites with high thermal conductivity via in-situ sintering of silver nanoparticles. Chem. Eng. J. **369**, 1150-1160 (2019). <https://doi.org/10.1016/j.cej.2019.03.150>
11. S. Rao, X. Zeng, X. Cheng, J. Fan, D. He et al., Damping, soft, and thermally conductive composite elastomer via introducing bottlebrush chains. Chem. Eng. J. **474**, 145847 (2023). <https://doi.org/10.1016/j.cej.2023.145847>
12. H. Niu, H. Guo, Y. Ren, L. Ren, R. Lv et al., Spherical aggregated BN/AlN filled silicone composites with enhanced through-plane thermal conductivity assisted by vortex flow. Chem. Eng. J. **430**, 133155 (2022). <https://doi.org/10.1016/j.cej.2021.133155>
13. M. Qin, Y. Huo, G. Han, J. Yue, X. Zhou et al., Three-dimensional boron nitride network/polyvinyl alcohol composite hydrogel with solid-liquid interpenetrating heat conduction network for thermal management. J. Mater. Sci. Technol. **127**, 183-191 (2022). <https://doi.org/10.1016/j.jmst.2022.04.013>
